# Lateral Curvature of the Spine

**Published:** 1898-08

**Authors:** Wirt A. Duvall

**Affiliations:** Baltimore


### LATERAL CURVATURE OF THE SPINE.

BY DR. WIRT A. DUVALL, A. M., M. D., BALTIMORE.

Lateral curvature of the spine is a permanent lateral deviation of the spinal column or a portion of it from the natural physiological direction. (Drachman.)

Accepting this very generally recognized definition of lateral curvature as true, it leaves a matter of conjuncture as to what is the physiological or correct spine. The perfect mortal (anatomically speaking, of course) we picture mentally after reading Gray, and exact measurements of human proportions we leave for writers of fancy and maiden ladies. Only those displacements which affect the health and happiness of the individual should receive the attention of the surgeon.

Of all the deformities probably none is more frequent than that of the lateral curvature of the spine. While age and sex, environment and general hygienic conditions, play their important part in the study of the malady, yet it attacks all, old and young, male and female, white and black. The young suffer more than the old. Statistics show about fifty-five per cent. of all cases before the twentieth year, and of this about eighty per cent. are females. What an appalling condition! The one individual that should be the nearest approach to perfection, anatomically and otherwise, the future white mother, is dangerously faulty as to her framework, and, too, to a very great extent. Her sister in black is almost free from this trouble.

Of the causes there are two of a general character, acquired and hereditary. To the acquired form I wish to invite your thought. Says one writer: "By far the greater number are acquired, and the predisposing cause is sex, girls being in the proportion of five to one boy." (Koelliker and Ketch.)

Why is this? Because girls wear corsets. This is the cause given by the late Dr. Atlee of Philadelphia. "Look at that interesting, delicate girl, pallid and wan, struggling wearily under her weight of clothing, which the strongest of her sex should not tolerate. All suspended from her shoulders? No, but upon her constricted waist."

Why is this condition? The dress habit, like the drug habit, is slow to fasten itself on, but hard to get rid of. Other causes, such as the faulty school desks, unfortunate daily occupation, but no cause, in the humble opinion of the writer, so prolific as the corset.

They act as splints to the backbone and thus weaken that which binds together the bony segments.

What is needed at all times is a backbone stiff enough to need no splinting. Throw away the corsets and tone up the ligament and muscles. To those of us who have had the privilege to listen to Tiffany these words are more familiar: "Remove the cause and put the part at rest." As a guide to the line of treatment, it can be said with equal emphasis, "Remove the corset and put the parts to work."

The normal spine is made up of a series of peculiar-shaped bones placed one upon the other and so relatively arranged as to form a protection for the delicate parts entrusted by nature to their care. This normal spine has its normal curves, and, too, for a purpose. They are the outpost.

The Silent Architect having arranged the column, with its curves, the maintenance of its integrity of continuity becomes a lasting duty. This can only be done by keeping as near to perfect health the strong bands that bind together these peculiar bones in their beautiful arrangement. Too much can not be said against those bits of apparel that prevent the proper employment of the spine, thus retarding a proper development. What follows in the train of a well-developed lateral curvature time does not admit for discussion now, but it can be said that there are many to-day the victims of disease that has for its able ally that which stands as one cause of lateral curvature—the corset.

Reckoning this bit of apparel as a great factor in the causation of this malady, we must charge the results of these displacements to this garment. Lateral displacements so change the relative positions of the bony segments of the spine as to encroach upon the lumen of the spinal canal, thus producing pressure upon the cord not only at the point of greater curvature, but at points distant therefrom. This pressure may be ever so slight, but the effects are present doubtless long before the clinical symptoms are manifest. While lateral curvature may not be charged with the production of all diseases, even unto toe-ache, it nevertheless does reduce the resisting power by faulty stimulation.

More attention should be given to dress reform in general, but a warfare should be waged against the corset till they are a thing of curiosity and an object of scorn. The healthy spinal column needs no splint; it needs activity, like the brain. Decay follows loss of motion. The elasticity of the ligaments is the best proof of this. Put off the splints and let the backbone get its natural stiffness through activity and health-giving motion. This done, and generations yet to be will rise up in thankful remembrance.—*Maryland Medical Journal.*

